# APBioNet's annual International Conference on Bioinformatics (InCoB) returns to India in 2018

**DOI:** 10.1186/s12864-019-5582-8

**Published:** 2019-04-18

**Authors:** Shandar Ahmad, Michael M. Gromiha, Gajendra P. S. Raghava, Christian Schönbach, Shoba Ranganathan

**Affiliations:** 1 School of Computational and Integrative Sciences, Jawaharlal Nehru University, New Delhi, 110 067 India; 2 Department of Biotechnology, Bhupat and Jyoti Mehta School of Biosciences, Indian Institute of Technology Madras, Chennai, Tamilnadu 600 036 India; 3 Centre for Computational Biology, Indraprastha Institute of Information Technology, Okhla Industrial Estate, Phase III, New Delhi, 110020 India; 4 Department of Biology, School of Science and Technology, Nazarbayev University, Astana, Kazakhstan; 5 International Research Center for Medical Sciences, Graduate School of Medical Sciences, Kumamoto University, Kumamoto, 860-0811 Japan; 6 Department of Molecular Sciences, Macquarie University, Sydney, NSW 2109 Australia; 7 Transformational Bioinformatics, Health and Biosecurity, Commonwealth Scientific and Industrial Research Organisation, Sydney, Australia

**Keywords:** InCoB, International conference on bioinformatics, APBioNet, Asia-Pacific bioinformatics network, APbians

## Abstract

InCoB, one of the largest annual bioinformatics conferences in the Asia-Pacific region since its launch in 2002, returned to New Delhi, India after 12 years, with a conference attendance of 314 delegates. The 2018 conference had sessions on Big Data and Algorithms, Next Generation Sequencing and Omics Science, Structure, Function and Interactions, Disease and Drug Discovery and Plant and Agricultural Bioinformatics. The conference also featured an industry track as well as panel discussions on Women in Bioinformatics and Democratization vs. Quality control in academic publishing. Asia Pacific Bioinformatics Interaction & Networking Society (APbians) was launched as an APBionet Special Interest Group. Of the 52 oral presentations made, 22 were accepted in supplemental issues of BMC Bioinformatics, BMC Genomics or BMC Medical Genomics and are briefly reviewed here. Next year’s InCoB will be held in Jakarta, Indonesia from September 10–12, 2019.

## Introduction

The International Conference on Bioinformatics (InCoB) was held for the first time in New Delhi, India in 2006 and returned in September 26–28, 2018 [1], with over 310 delegates. The Vice Chancellor of Jawaharlal Nehru University delivered the inaugural address. Three parallel sessions accommodated 24 sessions of 52 oral presentations while two evening sessions featured 154 poster presentations.

Fifteen keynote and invited talks addressed the current advances in genomics, transcriptomics, systems biology, protein structure and interactions, drug discovery, oncology and precision medicine. Three special sessions included an industry session with a presentation by Schrodinger Inc. and two panel discussions on Women in Bioinformatics and on Democratization vs. Quality control in academic publishing. At the Annual General Meeting of APBioNet on September 27, Christian Schönbach delivered the President’s Report on the state of APBioNet and its activities in the past year. The AGM was concluded with the announcement of election results for the Executive Committee (ExCo), term 2018–2020, by the returning officer. The list of elected ExCo members and office bearers is available at the APBioNet website [2].

## Manuscript submission and review

For the first time, submissions were handled directly on the Springer Nature Editorial Manager system. Authors submitted initial manuscripts on original research or software and database articles, for consideration to BMC editors to nominate one of four BMC journals for submission of: BMC Genomics, BMC Bioinformatics, BMC Systems Biology or BMC Medical Genomics. Selected manuscripts were then sent for peer review by the InCoB2018 Program Committee members. ~ 95% of the manuscripts sent for review were accepted after an average of 2 rounds of revision. Twenty-seven manuscripts were published in the InCoB2018 supplements of BMC Medical Genomics (2), BMC Genomics (6), BMC Bioinformatics (19) issues.

## Knowledge extraction

Extracting knowledge from large data collections, such as biological Big Data, has dominated bioinformatics endeavours in the past few years. With the focus on novel drug discovery, Lynn and his group have developed a Galaxy server to build a predictive model for QSAR-based virtual screening of small molecules [3], while Zheng at al. [4] have developed system to predict the side effects of approved drugs, based on negative samples. Pritam et al. [5] have identified epitope candidate against malaria based on a genome-wide immunoinformatics approach. Text-mining approaches have been used to mine published literature with BioReader [6] while Dhillon and co-workers have efficient setup clinical data management systems for breast cancer from electronic medical records [7]. Abu and coworkers [8] have developed an automated facial recognition system, using a minimal 3D feature set.

## Sequence analysis

With several large-scale genome sequencing projects of non-model organisms underway, the accuracy of the genome cannot be easily assessed, in the absence of a reference genome. For such projects, SQUAT [9] provides a rapid tool to evaluate de novo assemblies, rapidly identifying regions that are poorly mapped or require improvement. In the case of genome annotation, genes encoding small open reading frames are frequently missed. Mat-Sharani and Firdaus-Raih [10] have developed a generic annotation workflow for targeting these elusive regions and applied it successfully to a massive dataset of 31 fungal genomes.

Several novel methods for predicting sites involved in sumoylation [11], phosphoglyceration [12], glycation [13] and lysine glutarylation [14] from sequence data add valuable approaches to functional annotation of proteins. Since protein function is derived from structure, the functional characterization of intrinsically disordered proteins has remained difficult. Sharma et al. [15] have developed an excellent a support-vector machine (SVM) based approach, to identify functionally significant parts of such proteins, such as those involved in molecular recognition processes.

DNA methylation is a key epigenetic mechanism of genomic regulation. Large-scale epigenome analysis is critical for discovering novel therapeutic and prognostic biomarkers. Pappalardo and co-workers present EpiMethX [16] for such analysis, for discovering methylation hotspots, important for cancer progression. In ecological samples, on the other hand, there are multiple viral genomes, to be identified concurrently. To address this problem, Halgamuge and coworkers have developed a novel method, ENVirT [17] which is several orders of magnitude more accurate than other available methods.

## Transcriptome analysis

For clinical relevance, disease gene biomarkers need to be sub-type specific. Hsu et al. [18] have analysed gene expression profiles for two different types of glioma and determined subtype-specific candidate biomarkers relevant to survival. Raghava and his group have developed a carefully curated database for chromosomal fragile sites in the human genome, HumCFS [19], as a resource for genome instability analysis, relevant to cancers and neurological conditions.

MicroRNAs (miRNAs) are functionally important for gene regulation. Guo et al. [20] have sequenced miRNAs from an economic and horticulturally important plant, *Rosa rugosa* and report the functional enrichment miRNA profiles of leaves and petals.

In a pharmacogenomic approach, Taguchi [21] has analysed gene expression profiles of cells treated with different drugs to identify two pan-cancer candidate drugs, using a tensor decomposition-based unsupervised feature extraction approach.

## Structural bioinformatics

Changes in chromatin structures are associated with diseases, such as aggressive cancers. However, there is paucity of studies on 3D chromatin structure. Nagai et al. [22] report a complex relationship between chromatin reorganization at the structural level and gene regulation, with implications for cancer development.

Protein function is dependent on its 3D structure. Bioinformatics provides solutions to predicting the 3D structures of proteins that so not have experimental structures. Hardianto et al. [23] have used homology models of novel protein kinase C (PKC) isozymes to characterize their interaction with a candidate inhibitory drug molecule. On the other hand, Borah and Jha [24] have predicted an ab initio structural model for a pathogenic protein, HopS2, that affects tomato plants, to understand its pathogenic function, as a Type III secretion system.

For peptide-based therapeutics, it is important to know their exact binding sites on target proteins, using protein-peptide docking. As the majority of computational docking methods have been developed for drug-protein interactions, Agarwal et al. [25] have carried out extensive benchmarking of six most commonly used methods for a large number of protein-peptide combinations. They report that FRODOCK performed best in case of blind docking and ZDOCK in case of re-docking.

## Network analysis

Biological molecules interact and communicate with each other and such interactions are represented by networks. While this approach has led to several network representations of experimental data, some information is invariably missed. In order to detect hidden interactions, Elati and co-workers [26] have proposed a latent network approach for large-scale gene expression datasets, presenting impressive results on bladder cancer datasets. Manoj et al. [27] have analysed gene expression patterns sugarcane to identify genes that can withstand the effects of climate change and report specific genes which confer drought resistance and tolerance to salinity.

Sen et al. [28] have analysed structural networks to uncover the evolutionary changes in cancer relevant proteins. Kaalia and Rajapakse [29] have extended protein interaction networks to the entire human proteome and report enrichment of gene sets of known functions.

## Conclusion

With the growing interest in applying bioinformatics to personalized and precision medicine, InCoB is becoming increasingly important to the medical community and the 2019 conference will be hosted by Yarsi University in Jakarta, Indonesia, from Sept. 10–12, 2019.
